# Incidence of and Risk Factors for Central Venous Catheter Thrombosis: Results from a Single-Center Pediatric Intensive Care Unit

**DOI:** 10.3390/children11111394

**Published:** 2024-11-17

**Authors:** Maha Azzam, Yousef M. AlTalhi, Hani Alsawadi, Mohamed Humoodi, Abdullah Alzahrani, Amir Shehzad Hayat, Mohammed Bakhsh, Sara Osman

**Affiliations:** 1 Department of Pediatrics, King Abdulaziz Medical City, P.O. Box 65362, Jeddah 21556, Saudi Arabia; azzamma@ngha.med.sa (M.A.); attlhyyo@mngha.med.sa (Y.M.A.); zahraniab07@mngha.med.sa (A.A.); hayatas@mngha.med.sa (A.S.H.); bakhshmi@mngha.med.sa (M.B.); 2 King Abdullah International Medical Research Centre, Jeddah 21556, Saudi Arabia; 3 College of Medicine, King Saud bin Abdulaziz University for Health Sciences, P.O. Box 65362, Jeddah 21556, Saudi Arabia; 4 Pediatric Intensive Care Unit, Royal Hospital for Children, Glasgow G51 4TF, UK; mohamed.humood@ggc.scot.nhs.uk

**Keywords:** central venous thrombosis, deep vein thrombosis, PICC lines thrombosis

## Abstract

Background: Central Venous Catheter (CVC) is a necessary and important tool in managing acutely ill children and those needing complex care. CVC enables infusing venous medication, fluids, blood products, chemotherapy, total parental nutrition, and painless withdrawal of blood for laboratory testing when needed. Objective: To identify the incidence and risk factors for Central Venous Catheter-Related Thrombosis (CVC-RT) among patients admitted to the Pediatric Intensive Unit. Method: This was a prospective, observational, single-center study that was conducted over 17 months from September 2019 to January 2021 at King Abdulaziz Medical City, Jeddah. Design: Prospective observational study. Setting: King Abdulaziz Medical City, a tertiary care center in the western region of Saudi Arabia. Patients: Pediatric patients aged 1 to 168 months who were admitted to the PICU and required central line insertion (whether inserted centrally or peripherally) for more than 48 hours were included. Screening for thrombosis was performed within day 4–7 post-line insertion and again on the 14th day. Results: A total of 255 patients were enrolled over 17 months. The incidence rate of CVC-RT was 5.4%. The type of CVC was significantly different between the two groups; in the no thrombosis group, 59.2% had a central line while in the CVC-RT groups, 51.9% had a PIC line (*p* = 0.027). In a multivariate regression analysis including patients’ clinical profile, high D-dimer as baseline and low platelets were both significant risk factors for CVC-RT [adjusted OR = 3.22, CI (1.25–8.28), *p* = 0.015 and adjusted OR = 7.38, CI (2.18–25.02), *p* = 0.001], respectively. Conclusions: The current study found that PIC line was associated with an increased risk of CVC-RT, which is congruent with the literature. As children with CVC can have multiple risk factors for developing CVC-RT, it is important to conduct further large prospective studies to identify such factors and decrease the incidence of CVC-RT.

## 1. Introduction

Central Venous Catheter (CVC) placement is one of the most frequently performed procedures in patients admitted to Pediatric Intensive Care Units (PICUs) because it allows for the delivery of medications, IV fluids, blood products, and parenteral nutrition [1]. Catheter-related thrombosis is defined as thrombosis related to (DVT) that partially or completely occludes the vein in which the catheter line is inserted.

Venous obstruction, loss of vascular access and increased incidence of infections and pulmonary embolisms [2]. Currently, the prevalence rate of CVC-RT in children cannot be determined because multiple studies that yielded various ranges of 2–50% are attributed to differences in the populations studied and the methods used in diagnosing those diseases. These variations underline the necessity for the definition of certain actions that would help in the diagnosis of the complications and their treatment within the pediatric ward. As noted by [3], children undergo higher probabilities of developing CVC-RT for several reasons, and these include the use of a small vessel caliber in children, the medical conditions prevailing in children and prolonged use of CVC through intravenous feeding. Some studies with the identification of many aspects that can make childhood patients prone to developing CVC-RT are as follows: They include the kind of catheter used, the part of the body where the catheter was placed, whether the catheter has more than one opening or hole and the period the catheter was left in. Other causes can also be explained by factors such as cancer, infection, or coagulation disorder that the patient may be suffering from [4]. For example, it is possible to identify that children with hematologic malignancies are at an increased risk of developing CVC-RT because of the treatment and the process that pediatric patients have gone through. Moreover, thrombosis can be difficult to diagnose among children who have CVCs since many thrombotic episodes do not readily present themselves with typical symptoms. According to recent research [5], ultrasound is very useful in the diagnosis of thrombosis at an early stage in this group of individuals. The formation of thrombi is potentially dangerous, and early detection of the thrombi is necessary for intervention in patients’ care. Regarding the management of thrombosis, prevention has been reviewed and approaches such as anticoagulation, antibiotic-lock therapy, and catheter care have been discussed. However, the use of routine prophylactic doses of anticoagulation is still a matter of debate and there is great variability.

The purpose of this present study is to assess the incidence of CVC-RT in pediatric patients admitted to the PICU at King Abdulaziz Medical City, Jeddah and to also estimate the potential risk factors associated with it. The data contribute to developing different strategies for the prevention or reduction of thrombosis risk in dependence on the patients’ risk levels. Scientific research helps improve the practice of critical care practitioners by disclosing the evidence base behind CVC-RT. CVCs are useful in children who are critically ill, there is a chance of high-risk thrombosis that needs constant assessment and intervention. Thus, this study aims to complement the current literature by providing discrete concrete data on the pediatric population in a Middle Eastern tertiary care center and contributing to the global CVC-RT knowledge enhancement for children.

## 2. Methods

### 2.1. Study Design and Participants

This was a prospective observational study with a specific focus on Central Venous Catheter-Related Thrombosis, conducted over 17 months from September 2019 to January 2021 at King Abdulaziz Medical City, Jeddah. The research design aimed to identify the incidences and risk factors for CVC-RT among pediatric patients admitted to the PICUs. This study was conducted at King Abdulaziz Medical City, a tertiary care center located in the western region of Saudi Arabia. The hospital provides advanced medical care and has a well-established PICU equipped to handle critically ill pediatric patients requiring intensive monitoring and treatment. This study included pediatric patients aged 1 to 168 months who were admitted to the PICU and required central line insertion for more than 48 h. Both centrally and peripherally inserted central lines were considered. The option of central line versus PICC line, decided by the treating team, usually peripheral central catheters kept for those who require longer duration (more than two weeks). Patients were excluded if they required central line insertion for less than 48 h or had pre-existing thrombotic conditions. 

### 2.2. Sample Size Calculation

Sample size calculated based on the Yamane’s formula:

*n* = N/(1 + N(d)^2^)

where if *n* is the sample size, N is the population size, and d is the desired accuracy (0.05):

500 ÷ 1 + 500(0.05)^2^ = 222.

As per this formula, we need a sample size of 222 to achieve a statistically significant result. Our actual sample size was 255 patients in a period of 17 months.

### 2.3. Data Collection

Data were collected using a patient-recorded form containing demographic and clinical characteristics, including age, sex, primary diagnosis, and clinical factors such as the presence of limb asymmetry, edema, pain or redness. The CVC profile, including the indication for insertion (elective or emergency), type of CVC, number of lumens, number of insertion attempts, and duration of catheterization, was also documented. Screening for Thrombosis was performed within day 4–7 post-line insertion and again on the 14th day using ultrasound to detect any thrombus formation. Screening is considered earlier if symptoms and signs appear early, and this makes early detection of thrombosis more likely. The screening process included both clinical assessment and imaging. Baseline coagulation profiles, including platelet count, fibrinogen level, and D-dimer level, were assessed. Abnormalities in these parameters were noted and identified as potential risk factors for the development of CVC-RT. Given the variability in platelet counts across pediatric age groups, we ensured that higher reference ranges for infants (up to 600,000/µL) were considered. For consistency, the analysis follows the standard 150,000–450,000/µL range, unless otherwise noted.

### 2.4. Statistical Analysis

Data collected were analyzed using the statistical package for Social Sciences (SPSS) version 25.0 (IBM) for the summary of continuous variables into medians with interquartile ranges (IQR) as well as frequency and proportion for categorical variables. For continuous variables group with and without CVC-RT Mann–Whitney U test was applied, while for categorical variables Chi-square or Fisher’s exact test was applied. Further, the independent variables were analyzed using univariate logistic regression analysis to determine the risk factors associated with the development of CVC-RT. Potential confounders identified in univariate analysis with a *p*-value ≤ 0.05 were included in the multivariate logistic regression modelling to adjust for the effects of other variables. The results are presented in terms of adjusted odds ratios (ORs) and corresponding 95% CI of CVC-RT for risk factors. A *p*-value of <0.05 was regarded as statistically significant.

### 2.5. Ethical Considerations

The current study was reviewed and received approval from the Institutional Review Board (IRB) of King Abdulaziz Medical City. Each participant was required to provide signed informed consent from their parents or legal guardians before enrolling in this study. The methodology of this study complied with the Declaration of Helsinki and other standards of Good Clinical Practice.

## 3. Results

### 3.1. Patients’ Clinical Profile

A total of 255 pediatric patients requiring Central Venous Catheter (CVC) insertion were enrolled in this study over 17 months. The median age of the patients was 48 months, with an interquartile range (IQR) of 54 months in the no-thrombosis group and 110 months in the CVC-RT group, showing no significant difference (*p* = 0.295). The distribution of genders was similar in both groups, with males comprising 53.5% of the no-thrombosis group and 51.9% of the CVC-RT group (*p* = 0.870). The admitting diagnoses varied, with oncology patients constituting the largest group (50.9%) among those without thrombosis and 51.9% in the CVC-RT group. Other notable diagnoses included respiratory conditions, sepsis, and surgical cases, though none of these showed significant differences between the groups. Clinical factors such as limb asymmetry, edema, pain, and/or redness were significantly more prevalent in the CVC-RT group (55.6% vs. 6.6%, *p* < 0.001). Baseline coagulation profiles revealed that abnormal low platelets and high D-dimer levels were more frequent in the CVC-RT group (*p* < 0.001). D-dimer levels were measured in mg/L FEU (fibrinogen equivalent units) (Table 1).

### 3.2. Central Venous Catheter Profile (CVC)

The type of CVC used was significantly different between the groups. In the no thrombosis group, 59.2% had a central line compared to 29.6% in the CVC-RT group. Conversely, 51.9% of the CVC-RT group had a PIC line compared to 27.6% in the no thrombosis group (*p* = 0.011). The number of lumens and insertion attempts did not show significant differences between groups. The median duration with a line was longer in the CVC-RT group, although not significantly so (30 days vs. 22 days, *p* = 0.087) (Table 2).

### 3.3. Thrombosis Screening

Screening for thrombosis was performed at two intervals, day 4–7 and day 14 post-line insertion. On days 4–7, 11 patients (4.8%) screened positive, and of these, 2 (19%) were confirmed with thrombosis via ultrasound. On the 14th day, one patient (0.39%) screened positive, who was subsequently confirmed to have thrombosis (Table 3).

### 3.4. Univariate Analysis of Risk Factors

Univariate logistic regression analysis identified several factors associated with the development of CVC-RT. Low baseline platelets (OR = 2.49, 95% CI 1.01–6.15, *p* = 0.043) and high baseline D-dimer levels (OR = 5.53, 95% CI 1.70–17.96, *p* = 0.002) were significant risk factors. PIC-line use was also significantly associated with CVC-RT (OR = 2.82, 95% CI 1.26–6.33, *p* = 0.014), whereas central-line use was associated with a lower risk (OR = 0.29, 95% CI 0.13–0.66, *p* = 0.003) (Table 4).

### 3.5. Multivariate Analysis

Multivariate logistic regression confirmed that high baseline D-dimer levels (adjusted OR = 6.73, 95% CI 2.03–22.27, *p* = 0.002) and PIC-line use (adjusted OR = 3.40, 95% CI 1.34–8.63, *p* = 0.010) were independent risk factors for CVC-RT, while central-line use remained protective (adjusted OR = 0.25, 95% CI 0.10–0.65, *p* = 0.004) (Table 5). Upon re-running the multivariate analysis with the inclusion of high D-dimer levels, low platelet counts, and PIC-line usage, the adjusted odds ratios were recalculated as follows.

## 4. Discussion

The research findings present a descriptive assessment of the CVC-RT in pediatric patients according to their demography, clinical characteristics, and potential risks. The results support that children requiring CVCs need to be closely observed and deliberate decisions made in their intensive care [6]. With regards to age and sex, the study population was in a relatively moderate range and gender does not appear to contribute to CVC-RT predisposition. The median age of the patients is approximately 48 months, which supports earlier findings that both very young children and older children may develop CVC-RT. In a study done by Moiz and his group, they found that adolescents were more likely affected by sinus thrombosis while infections was a risk factor for all age groups [7]. The gender distribution with slightly more male patients is in line with the findings reported in our work, showing increased thrombotic events in male patients. In one of the studies by [8], as compared to the control group, the CVC-RT group exhibited more clinical indicators of CVC that included limb asymmetry, edema, pain, and redness. Such signs, which are representative of thrombotic occurrences, highlighted the need for frequent clinical checks to detect thrombosis in its early stages. The several-fold higher frequency of RL, as well as the D-dimer level in the CVC-RT group highlights the usefulness of these hematological indices as indicators of thrombosis [9].

In this paper we highlighted the role of the independent variable as it influenced the risk of developing thrombosis. When we applied univariate logistic regression analysis, we discovered that factors like low baseline platelets count and high D Dimer put the patient at high risk of developing CVC-RT (OR = 2.49, 95% CI 1.01–6.15, *p* = 0.043) for low platelet count (OR = 5.53, 95% CI 1.70–17.96, *p* = 0.002) for high baseline D-dimer levels. PIC-line use was also significantly associated with CVC-RT (OR = 2.82, 95% CI 1.26–6.33, *p* = 0.014), whereas central-line use was associated with a lower risk (OR = 0.29, 95% CI 0.13–0.66, *p* = 0.003. In the same regard, multivariate logistic regression confirmed that high baseline D-dimer levels and low baseline platelets count were independent risk factors for CVC-RT, with a higher odds ratio and statistically significant *p*-value while central-line use remained protective (adjusted OR = 0.25, 95% CI 0.10–0.65, *p* = 0.004).

In contrast to our finding, a systematic review with a meta-analysis performed by Patrícia Cristina and her group looked for the association of biomarkers and DVT in patients using PICC and found, through looking at 28 studies that PICC-related CVC-RT was associated with higher D-dimers (0.37 g/mL, 95% CI 0.02, 0.72; *p* = 0.04, I2 = 92%; *p* for heterogeneity < 0.00001) and with higher platelets (8.76 × 10^9^/L, 95% CI 1.62, 15.91; *p* = 0.02, I^2^ = 41%; *p* for heterogeneity = 0.06) [10]. Furthermore, a study conducted by a group of Chinese researchers revealed no relationship among platelets, D-dimer, and PICC thrombosis [11].

The use of PIC lines was found to be closely related to the possibility of thrombosis more than the use of central lines [12] and it was noted that PICCs are prone to thrombotic events, mainly due to the small and long size of the catheter, which may cause endothelial damage and blood clot formation. In regard to this, there may be associated advantages of central lines, which may be attributed to the larger ability and small size of the lines that translated to increased blood flow and reduced endothelial activation [13]. Interestingly a Multivariate logistic regression done by Alhazmi SM and her group revealed that central venous catheters had the highest risk for central line associated blood born infections in comparison with other types of lines(odds ratio: 10.088; 95% CI = 2.595–39.215; *p* = 0.001) [14]. Thrombosis screening is performed at different times after the CVC insertion, with evidence [15] showing that using ultrasound for early detection helped identify thrombotic incidences. The need for invasive procedures to obtain confirmed diagnoses can be reduced by the high positive predictive value of ultrasound screening by day 14 for detection of CVC-RT (as shown in our results). Based on these findings, researchers [16] called for the adoption of standard screening using ultrasound routinely to improve the early diagnosis and treatment of CVC-RT. From the univariate and multivariate analysis, the independent predictors of CVC-RT included high baseline D-dimer levels and the use of a peripheral IV catheter accessed through the internal vein with an acupuncture technique [17]. Symptoms of active fibrinolysis or hyper fibrinolysis have been shown in several case-control and prospective studies to be correlated with thrombotic events and constitute an established risk factor for thrombosis [18]. PIC lines linked with higher thrombosis risk require more consideration and probably opting for central lines in clients at high risk of thrombosis.

Some limitations include that this study was a center-based approach and has fewer comparisons in the thrombosis group due to the small sample size, which may reduce the generalization of the findings. Further research should involve larger groups of participants to replicate such results and identify other potential risk factors. Furthermore, research on the effectiveness of different prophylactic measures to prevent thrombosis in pediatric patients with CVC might be helpful when addressing the issue of CVC-RT. Notwithstanding these limitations, the outcomes of this research possess several clinical implications. Firstly, the patients’ hematological parameters should be considered to identify candidates for high levels of thrombosis upon CVC insertion. Secondly, when it comes to CVC type selection, the patient’s risk should be considered, and using central lines is recommended for patients with high risks. Thirdly, it is necessary to build a simple ultrasonic examination for thrombosis as a regular follow-up examination for patients with CVCs.

## 5. Conclusions

In conclusion, the research that has been performed showed that some factors are essential in children suffering from CVC-RT, and these include hematological indexes, type of CVC, and regular check-ups. Integrating these findings into clinical practice has the potential to improve patient outcomes by limiting thrombosis in pediatric patients in need of CVC.

This study adds to the knowledge on this topic as we looked for biomarkers and CVC-RT in patients with PICC.

Our result is different from the current literature data, so it is a novel result and serves as a trigger for more robust studies that aim to determine the relationship between hematological indexes and CVL-RT.

## Figures and Tables

**Table 1:** Demographics and clinical profile.

Variables	Total Patients with CVC = 255		*p*-Value *
	No CVC-RT	CVC-RT	
	*n* = 228 (%)	*n* = 27 (%)	
Median age in months (IQR)	48 (54)	48 (110)	0.295
Sex (%) Male Female	122 (53.5) 106 (46.5)	14 (51.9) 13 (48.1)	0.870
Admitting diagnosis (%) Oncology patient Respiratory patient Sepsis Surgical patient Others **** Cardiac patient	116 (50.9) 6 (2.6) 72 (31.6) 8 (3.5) 17 (7.5) 9 (3.9)	14 (10.8) 1 (3.7) 9 (33.3) 1 (3.7) 8 (7.4) 0	0.945 **
Clinical factors **** (%) Yes	15 (6.6)	15 (55.6)	<0.001 **
High-risk factor present (%)	58 (25.4)	4 (14.8)	0.224
Already on anti-coagulant therapy at line insertion (%)	10 (4.4)	2 (7.4)	0.483
Baseline coagulation profile (%) Normal Abnormal low platelets Abnormal high fibrinogen Abnormal high D-dimer All parameters abnormal	167 (73.2) 33 (14.5) 4 (1.8) 9 (3.9) 15 (6.6)	9 (33.3) 8 (29.6) 1 (3.7) 5 (18.5) 4 (14.8)	<0.001 **

CVC: Central Venous Catheter; CVC-RT: Central Venous Catheter-Related Thrombosis; IQR: interquartile range. * Using Chi-square or Mann–Whitney test with a 95% confidence interval. ** ≥25% of cells had an expected count of less than 5. This included patients with trauma, metabolic, or neurological emergencies. **** Limb asymmetry, oedema, pain, and/or redness. Normal platelet count (150,000–450,000 platelets per microliter of blood). D-dimer is considered less than 0.5 mg/L FEU(fibrinogen equivalent unit) as the standard cut-off for thromboembolism. Normal fibrinogen level (2.0 to 4.0 g/L).

**Table 2:** The difference in the Central Venous Catheter (CVC) profile and laboratory markers between patients who developed CVC-related thrombosis (CVC-RT) and those who did not.

Variables	Total Patients with Central Line = 255		*p*-Value ^¶^
	No CVC-RT	CVC-RT	
	*n* = 228 (%)	*n* = 27 (%)	
Indication Elective Emergency	197 (86.4) 31 (13.6)	26 (96.3) 1 (3.7)	0.142
Type of CVC Central line Implantable lines * PIC line	135 (59.2) 30 (13.2) 63 (27.6)	8 (29.6) 5 (18.5) 14 (51.9)	0.011
The overall number of line lumens One Two Three	40 (17.5) 87 (38.2) 101 (44.3)	3 (11.1) 16 (59.3) 8 (29.6)	0.107
Number of attempts (%) One time Two times >Two times	42 (18.4) 134 (58.8) 52 (22.8)	1 (0.4) 17 (63) 9 (33.3)	0.117 **
Median duration with a line in days (IQR)	22 (15)	30 (12)	0.087
Median duration till thrombosis occurs in days (IQR)	22 (15)	30 (12)	Not Applicable ***

PIC line: peripherally inserted central line; IQR: interquartile range. ^¶^ Chi-square. * Porta Cath/Hickman/Proveac. ** One cell (16.7%) has an expected count of less than 5. The minimum expected count is 4.55. *** There were many outliers in the thrombosis group.

**Table 3:** Central Venous Catheter-Related Thrombosis (CVC-RT) screening details.

Thrombosis	Total Patients with CVC = 255	
	Screening status	Positive Official US
Day 4–7 Screening		
Negative	197 (77.3)	Not required
Positive	11 (4.8)	2 (19% out of 11)
Not done/Not applicable	20 (4.3)	-
Day 14 Screening		
Negative	104 (40.8)	Not required
Positive	1 (0.39)	1 (100% out of 1)
Not done/Not applicable	123 (48.2)	-

**Table 4:** Univariate regression analysis of independent variables affecting/predicting the development of Central Venous Catheter-Related Thrombosis (CVC-RT).

Variables	Developed CVC-RT *n* = 27 (%)	Odds Ratio (CI)	*p*-Value
Sex (female)	13 (48.1)	1.07 (0.48–2.38)	0.870
Age ≤ 1 year	5 (18.5)	0.98 (0.35–2.73)	0.966
Age ≤ 2 years	7 (25.9)	0.74 (0.30–1.84)	0.519
Age ≥ 6 years	10 (37)	1.20 (0.52–2.75)	0.666
Sepsis as the primary diagnosis	9 (33.3)	1.08 (0.46–2.53)	0.853
Oncological as the primary diagnosis	14 (51.9)	1.04 (0.47–2.31)	0.924
High risk	4 (14.8)	0.51 (0.17–1.54)	0.224
Low platelets as a baseline	8 (29.6)	2.49 (1.01–6.15)	0.043
High D-dimer as a baseline	5 (18.5)	5.53 (1.70–17.96)	0.002
High fibrinogen as a baseline	1 (3.7)	2.5 (0.23–20.0)	0.490
Central line	8 (29.6)	0.29 (0.12–0.69)	0.003
PIC line	14 (51.9)	2.82 (1.26–6.33)	0.014
Implantable line	5 (18.5)	1.50 (0.53–4.26)	0.444
1-lumen line	3 (11.1)	0.59 (0.17–2.05)	0.399
2 or 3-lumens line	24 (88.9)	1.70 (0.49–5.93)	0.399

**Table 5:** Multivariate analyses for risk factors/predictors associated with central-line-related thrombosis (CVC-RT) (using platelet count and D-dimer).

Variables	Total Patients with Central Line = 255		Crude Odds Ratio (CI)	*p*-Value	Adjusted Odds Ratio (CI)	*p*-Value
	No CVC-RT *n* = 91	Developed CVC-RT *n* = 25				
Clinical Profile Model						
Low platelets as baseline	33 (14.5)	8 (29.6)	2.49 (1.00–6.15)	0.043	3.22 (1.25–8.28)	0.015
High D-dimer as baseline	9 (3.9)	5 (18.5)	5.53 (1.70–17.96)	0.002	7.38 (2.18–25.02)	0.001

## Data Availability

The original contributions presented in this study are included in this article. Further inquiries can be directed to the corresponding author.
